# Does WeChat use intensity influence Chinese college students' mental health through social use of WeChat, entertainment use of WeChat, and bonding social capital?

**DOI:** 10.3389/fpubh.2023.1167172

**Published:** 2023-11-24

**Authors:** Mengfan Xia, Jing Liu

**Affiliations:** School of Public Administration, Hohai University, Nanjing, China

**Keywords:** WeChat usage intensity, WeChat use motivations, bonding social capital, mental health, Chinese college students

## Abstract

**Background:**

Previous research notes that the usage of WeChat is significantly related to individuals' mental health, but the underlying mechanism is still not completely discovered. The present study aimed to explore the sequential mediating roles of WeChat use motivations and bonding social capital on the effects of WeChat use intensity on mental health in Chinese college students.

**Method:**

The present study adopted an online survey with a total of 487 Chinese college students. Correlation analysis and serial mediation analysis were measured by process regarding the hypothesis.

**Results:**

The study presented findings indicating that WeChat use intensity had both direct and indirect impacts on the levels of life satisfaction and loneliness experienced by college students in China. Specifically, the utilization of WeChat for social motivation and entertainment motivation was found to have a suppressive effect on the relationship between the intensity of WeChat usage and individuals' life satisfaction. The association between the intensity of WeChat usage and mental health outcomes (life satisfaction and loneliness) was found to be mediated by bonding social capital. Furthermore, the association between the intensity of WeChat usage and mental health was found to be mediated by the sequential mediation effects of using WeChat for social motivation and bonding social capital, as well as the sequential mediation effects of using WeChat for entertainment motivation and bonding social capital.

**Conclusion:**

Our findings provide implications for policymakers and social workers regarding renovating the perceptions of the relationships between WeChat use intensity and overall mental health. Specifically, practical online activities and services of SNSs are recommended to be designed for meeting social and recreational gratifications and boosting bonding social capital, which in turn promotes psychological wellbeing.

## 1 Introduction

WeChat (微信; Wexin) is a highly popular social networking site (SNS) that was launched by Tencent Technology in January 2011 (1). Due to the blocking of popular social media platforms such as Facebook, YouTube, and Twitter in China, marketers have turned to WeChat as a viable means of direct communication with their Chinese target audience (2, 3). WeChat is a versatile program that extends beyond the conventional features of social networking services (4). WeChat, a major messaging platform, shares similarities with WhatsApp. It was originally created to enhance the accessibility and efficiency of online communication. WeChat offers several features such as text and voice messaging, instant message notifications, and video/voice calling capabilities (5). To cater to the diverse needs of its users, the WeChat team has devised a range of social and entertainment services. These include Moments, which allows users to share photos and videos; WeChat Group, which enables group conversations; Shake, which facilitates the discovery of other users currently engaged in the Shake feature; People Nearby, which enables users to locate others in close proximity; and Subscriptions, which provides users with access to subscribed articles (6). According to Liao et al. (7), WeChat has more than 1.2 billion monthly active users who have accessed WeChat through their smart mobile phones and computers since 2021. Given the increasing prominence of WeChat in everyday life, recent scholarly investigations have focused on examining the influence of WeChat usage on individuals' social networks and mental wellbeing (6, 8). Several studies have demonstrated a positive correlation between the intensity of WeChat usage and the social and psychological consequences for individuals (9–11). In line with concerns expressed by scholars on the negative consequences associated with Facebook and other global social networking sites (SNSs), some researchers express apprehension about the adverse impacts attributed to WeChat. Specifically, various studies have focused on the emergence of technology-related disorders, such as addiction to mobile phones (12), Internet usage (13), computer and video games (14), negative body image (15), and mental disorders (2, 16). According to Williams (17) assertion, how researchers see and define the medium can influence the development of cyber-pessimism and cyber-optimism. The continuous debate between cyber-pessimists and cyber-optimists is notwithstanding, and there exists a subject of more scholarly significance: the examination of how WeChat, specifically, exerts a positive influence on the physical and mental wellbeing of its users. This study sought to examine the underlying mechanisms between the usage of WeChat and mental health, in line with mutual concerns in this area.

**Figure 1:**
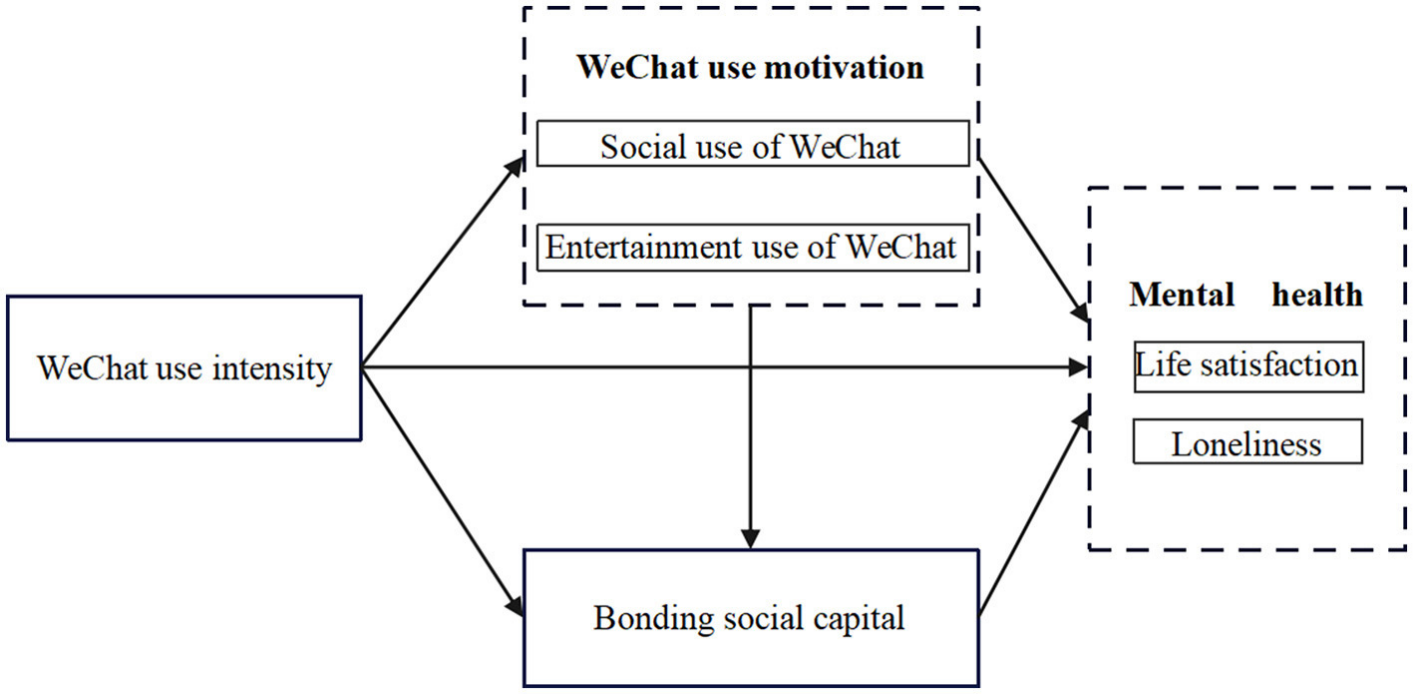
Hypothesized model of the impact of WeChat use intensity on mental health among college students in China: WeChat use motivations and bonding social capital as mediators. Age, gender, and education level as control variables.

To the best of our knowledge, previous studies only identified the isolated mediation role of WeChat use motivation or social capital, but there is no research systematically involving the potential mediators regarding examining the mechanisms between WeChat use intensity and mental health. Pang (18) designed the study to reveal the full mediation effect of social capital on the effect of WeChat use intensity on the subjective wellbeing of Chinese international students in Germany, but WeChat use motivation was not involved in his study. In addition, the research is aimed at foreign students in China. Due to cultural differences, the impact of WeChat use on the mental health of college students in China may be inconsistent. In contrast, Wen et al. (19) suggested that WeChat use motivation significantly mediates the association between WeChat use intensity and mental health. However, they did not investigate the roles of bonding social capital. To fill the current gap, the current research focused on exploring the underlying associations between WeChat use intensity and mental health, by introducing WeChat use motivation and bonding social capital as sequential mediators simultaneously. Regarding WeChat use motivation, we simultaneously consider the social use of WeChat and the entertainment use of WeChat. We also simultaneously consider two main types of mental health: life satisfaction and loneliness. Meanwhile, our study may expand the framework of Pang and Wang (20) into two important aspects. First, this is the first research that integratively and systematically examines the relationship between WeChat use intensity, motivations, bonding social capital, and mental health. By investigating the mediating influence of WeChat use motivation and bonding social capital, this research offers a more thorough comprehension of the mechanisms underlying WeChat use intensity in Chinese college students' mental health. Second, from the perspective of self-determination theory and the emotional motivation theory, this article also assists college students' proper and scientific use of new media which may exert positive effects on their psychological wellbeing. That is to say, these findings may guide the development of prevention and intervention strategies to protect college students from improper WeChat use. The theoretical models of this study are illustrated in Figure 1.

## 2 Literature review and research hypotheses

### 2.1 The mediating roles of WeChat use motivation

The evaluation of WeChat usage intensity frequently centers on the behavioral aspect, especially analyzing individuals' daily usage patterns. This assessment incorporates multiple indications, such as the quantity of WeChat friends, the duration of WeChat usage, and the frequency of platform access. According to the findings by Wen et al. (19), the mere utilization of WeChat does not intrinsically elicit emotions of contentment or unhappiness among individuals. However, the users' ultimate emotional experiences predominantly arise from the reasoning and methodology applied in employing the platform. Motivation exerts a substantial impact on persons' behavior and their entire emotional experiences. The use and gratification theory [UGT; (21)] has become increasingly popular in the study of motives within the media domain, particularly in relation to social networking sites (SNSs) (22–24). According to Blumler and Katz (21), the UGT framework suggests that individuals engage in an active process of selecting social media sites to meet their specific needs. According to Leung and Wei (25), the anticipation of continuing can be influenced by several forms of motives, which can be predicted based on patterns of usage. Through the implementation of the Unified Theory of Acceptance and Use of Technology (UGT), researchers can systematically assess the extent to which social networking site (SNS) usage contributes to the facilitation of incentives that are specific to SNS platforms. WeChat is a versatile application that offers a range of routine services. It has gained recognition as a highly user-friendly social networking site (SNS). WeChat is committed to developing localized functions, such as WeChat Moments, WeChat Payment, and online purchases, which are not available on other Chinese and international SNS platforms. WeChat's competitive advantage in the Chinese social networking sites (SNSs) industry can be attributed to its exclusive offerings, which encompass a diverse range of features not readily available on alternative platforms. The voluntary orientations of users contribute to the increased consumption of specific media (26) and the gratifications derived from using particular services. Consequently, individuals who have experienced satisfaction through WeChat are inclined to develop a reliance on the platform, regularly seeking and obtaining gratification exclusively through WeChat. Motivational factors mostly consist of gratifications. Moreover, the empirical research conducted on WeChat aligns with the principles and guidelines of UGT. According to Wen et al. (19), the level of intensity in using WeChat can serve as an indicator of several motivations for the use of the platform. According to the study conducted by Montag et al. (27), it was discovered that regular use of WeChat has the potential to influence particular motives. These motivations subsequently prompt individuals to utilize WeChat as a means of fulfilling their psychological needs. It can be argued that the cultivation of motivation in the use of WeChat is possible, as frequent usage does not necessarily lead to increased boredom among users. Instead, it has the potential to strengthen a user's motivation to continue using the platform and elicit good emotions. According to self-determination theory, intrinsic motivation ensures the activity is performed for its enjoyment rather than for external outcomes and is associated with a multitude of positive outcomes (28). Intrinsic motivation flourishes in contexts where the three psychological needs, competence, autonomy, and relatedness, are satisfied (29).

WeChat use motivation may act as a mediator between WeChat use intensity and mental health. Numerous scholars believe that SNSs are neither good nor bad, but the types of utilization matter (30, 31). WeChat use itself does not bring happiness or unhappiness to people, while the user's ultimate emotional experience mainly comes from the reason and method of use. Use motivation plays an important role in use behaviors and the overall emotional experience (19). In the era of social media, several scholars have applied the UandG approach to discover the psychological motivations for using social networking sites. The previous research identified the important motivations underlying the use of the Internet including information, convenience, entertainment, and social interaction (32). Based on the self-determination theory, Ryan and Connell defined four types of reasons for behaviors: external reasons (external authority or fear of punishment), introjected reasons (internal, esteem-based pressure, e.g., shame), identification reasons (values or goals), and intrinsic reasons (enjoyment or fun inherent to the behaviors) (33). Guo et al. (34) deprived “social-informational function” and “entertaining-recreational function” from Weiser's (35) Internet Use Function Scale to assess WeChat-oriented motivations. Social use of SNS and entertainment use of SNS are two prominent motivators that have been examined by many studies (36–39). Therefore, this study focuses on investigating social use motivation and entertainment use motivation. Social use of WeChat denotes individuals use WeChat for aims of interpersonal communication, social interaction, and social presence, while entertainment use of WeChat focuses on the motivations of enjoyment and passing the time (34, 40).

A majority of SNS studies report that social and entertainment motivations exert significant effects on subjective wellbeing (18, 41). Based on self-determination theory, SNS media can provide specific satisfaction for individual users. The social utilization of social media platforms has been found to have the potential to enhance social support, reinforce an individual's connection to the physical world, and facilitate self-expression and the acquisition of social support (42). Furthermore, Guo et al. (34) found that entertainment-recreational functions of SNS could contribute to the aggravation of loneliness, which is in line with early SNS research that entertainment use of technological devices (e.g., digital games) tends to cause addictive behaviors and mental illnesses (43). In contrast, Poppelaars et al. (44) found that a video game, being promoted as simply fun, likely appeals to a person's intrinsic values making it less likely to provoke reactance. Wen et al. (19) found that intrinsic use motivation (e.g., use of WeChat for fun) was the mediator between the use intensity and subjective wellbeing, while the other three types of motivation (external, introjection, and identification) cannot predict subjective wellbeing significantly. According to Wang et al. (45), the social-interactive use was positively related to college students' subjective wellbeing, but the entertainment was not. The ambiguous outcomes may be attributed to inconsistent measurements for entertainment motivation, in particular, the definition of “entertainment.” For example, Li et al. (46) measured “entertainment” from three dimensions, namely, fantasy, escapism, and enjoyment, while Xu et al. (47) measured “entertainment use” from affection and leisure. To avoid bias caused by inconsistent measurements, the present study adopted Guo et al. (34) methods of assessing social and entertainment motivations. Therefore, this study recommends the hypothesis as follows:

Hypothesis 1a: Social usage mediates the effect of WeChat use intensity on life satisfaction.

Hypothesis 1b: Social usage mediates the effect of WeChat use intensity on loneliness.

Hypothesis 2a: Entertainment usage mediates the effect of WeChat use intensity and life satisfaction.

Hypothesis 2b: Entertainment usage mediates the effect of WeChat use intensity and loneliness.

### 2.2 The mediating role of bonding social capital

Bonding social capital could potentially serve as a mediator in the relationship between the level of WeChat usage and an individual's mental wellbeing. Social capital can be described as a resource within an ecological context, encompassing elements such as value, connection, and trust. This resource is found within the social networks of individuals, with a particular emphasis on social networking sites (SNSs) (48, 49). In his seminal study, Putnam (50) initially introduced the notion of bonded social capital. According to Putnam (50) conceptual framework, bonding social capital is cultivated through the establishment of close-knit networks, wherein individuals can both offer and receive emotional support within their social connections. According to Chen and Li (51), social media possesses the capacity to bring about changes in individuals' wellbeing through major mediators, rather than exerting a direct influence, such as the formation of bonded social capital. WeChat functions as a communication platform that primarily supports interactions among individuals and small groups. The primary objective of this platform is to enhance interpersonal connections and cultivate a sense of confidence among individuals who are already familiar with one another, such as relatives and coworkers (52). Furthermore, the establishment of stronger interpersonal ties can be facilitated by participating in intimate self-disclosure, which involves the communication of private and in-depth information at a personal level. This concept aligns with the social penetration theory put forth by Altman and Taylor (53). Hence, it is apparent that engaging in individualized conversations on WeChat yields advantages in the development of social capital, which in turn facilitates the establishment of social bonds. Numerous research studies have been conducted to investigate the impact of social networking site (SNS) usage on the dynamics of bonding social capital, as evidenced by the works of Burke et al. (54), Kwon et al. (55), Papacharissi and Mendelson (56), and Raacke and Bonds-Raacke (57). Valenzuela et al. (41) provide a more detailed elucidation. According to previous research conducted by Alsaggaf (58), McQuail (59), and Valenzuela et al. (41), it has been proposed that the utilization of social networking sites (SNSs) has the potential to satisfy individuals' desires for social integration and personal identity presence. These factors have been highlighted as important indicators of social capital at the individual level. According to Pang (18), research on WeChat usage has identified a significant correlation between the intensity of WeChat use and the development of bonding social capital. Drawing upon existing scholarly works, the current research posited a hypothesis on the substantial and positive correlation between the level of engagement with WeChat and the development of bonding social capital.

Since the year 2001, mental health has been recognized and acknowledged as a significant worldwide burden of disease by the WHO. Scholars have placed significant emphasis on the importance of bonding social capital in mitigating psychological diseases. This emphasis is driven by the objectives of fostering trust, safeguarding distinct social identities, and facilitating reciprocal interactions among individuals (50, 60). Extensive scholarly inquiry, both theoretical and empirical in nature, has been dedicated to exploring the association between bonding social capital and mental health or subjective wellbeing (61–64).

The increasing recognition of the importance of social capital has led to a surge of research investigating the impact of bonding social capital on individuals' levels of life satisfaction and feelings of loneliness. Diener et al. (65) suggest that self-rated life satisfaction serves as an indicator of an individual's overall happiness with various life occurrences. The presence of low life satisfaction is associated with an increased likelihood of experiencing subjective wellbeing concerns, as evidenced by studies conducted by Fergusson et al. (66) and Swami et al. (67). According to the findings of Elgar et al. (60), which were derived from a sample of 69,725 adults across 50 nations, it was proposed that there exists a direct and indirect relationship between bonding social capital and the health and life satisfaction of individuals. The findings of two research conducted by Pang (18, 68) indicate that bonding social capital, formed through the WeChat social network, is a significant predictor of life satisfaction. According to Weiss (69), the construct of loneliness can be characterized as a state of social and emotional isolation, rather than being indicative of personal inadequacy. Loneliness has been found to exacerbate the negative effects associated with low subjective wellbeing, as indicated by pertinent research (70). In their study, Burke et al. (71) analyzed self-rated Facebook data from individuals of several nationalities. Their findings indicate a strong and inverse relationship between bonding social capital and feelings of loneliness. Nyqvist et al. (72) discovered that low trust, which is a fundamental element of bonding social capital, serves as a strong indicator of feelings of loneliness. According to Pang (18), research on WeChat usage indicates that bonding social capital is a significant factor that has an inverse relationship with feelings of loneliness. Consistent with other research, the present study posited that bonding social capital is a significant predictor of both life happiness and loneliness. Therefore, the essay presents the following assumptions:

Hypothesis 3a: Bonding social capital mediates the effect of WeChat use intensity and life satisfaction.

Hypothesis 3b: Bonding social capital mediates the effect of WeChat use intensity and loneliness.

### 2.3 The relationship between WeChat use motivations and bonding social capital

Previous studies have conducted empirical studies on Social Networking Sites (SNS), specifically focusing on platforms such as Facebook. These studies have identified that incentives related to social interaction and amusement might serve as predictors for the accumulation of social capital (55, 73). According to Procentese et al. (42), the utilization of social media platforms has been found to enhance social support, foster interpersonal connections in the physical realm, and facilitate self-expression and social support. Pang (9) asserts that the cultivation of strong connections via social media platforms has the potential to enhance the significance of international students and augment their coping mechanisms, namely in terms of social support and social capital. According to the findings of Jin and Zhang (74) study, various aspects of WeChat usage, such as the number of China friends on WeChat, information sharing, social interaction, and WeChat dependence, have a positive correlation with social adaptation. Additionally, qualitative research indicates that the social network formed through WeChat contributes to the improvement of individuals' social skills, alleviation of loneliness, and development of a new cultural identity. Pang (18, 68) conducted a pair of interconnected studies, which demonstrated a substantial positive correlation between the duration of WeChat usage and the extent of one's WeChat network with increased levels of bonding social capital. Hence, drawing upon the UGT and existing empirical studies, it is reasonable to posit that the motivation to use WeChat serves as a mediating factor in the relationship between the intensity of WeChat usage and the formation of social capital. Hence, this analysis posits the subsequent assumptions:

Hypothesis 4a: WeChat use intensity exerts indirect effects on life satisfaction through the social use of WeChat and bonding social capital.

Hypothesis 4b: WeChat use intensity exerts indirect effects on loneliness through the social use of WeChat and bonding social capital.

Hypothesis 5a: WeChat use intensity exerts indirect effects on life satisfaction through the entertainment use of WeChat and bonding social capital.

Hypothesis 5b: WeChat use intensity exerts an indirect effect on loneliness through the entertainment use of WeChat and bonding social capital.

## 3 Methods

### 3.1 Data collection and participants

In this section, we will discuss the methods used for data collection and provide information about the participants included in the study. The study was conducted between May and June 2023, employing a multi-stage cluster random sampling technique. Initially, a random selection process is employed to designate four districts within the city of Nanjing, located in China. Subsequently, a university is selected at random within each district. In each educational institution, a single class is randomly selected from a specific major, spanning from the freshman through senior levels. The survey sample for this study comprises all students within each specified class. The researchers acquired consent letters from the subjects. A total of 501 questionnaires were collected for this study, facilitated by class counselors using an online data-collecting platform called SOJUMP (http://www.sojump.com). SOJUMP is a professional and extensively utilized online survey platform in China, boasting a vast membership base of millions of registered individuals around the nation (9). Furthermore, several studies (6) have utilized SOJUMP to investigate individuals' WeChat usage patterns and behaviors. The survey approach was used due to its suitability for the current study, which focuses on examining the potential impact of WeChat usage intensity on individuals' mental wellbeing within mobile social networking service contexts. Moreover, employing an online survey is the most economically viable and temporally expedient approach for gathering data from a substantial cohort of individuals. After eliminating surveys that were deemed invalid due to frequent responses, contradictory positive and negative alternatives, completion times of <120 s, and missing or erroneous information, a total of 487 valid questionnaires were received, resulting in an effective response rate of 97.2%. The sample consists of 246 individuals who identify as men and 241 individuals who identify as women. The mean age of the sample is 20.09 years, with a standard deviation of 1.42. The student population consists of 159 first-year students, 110 second-year students, 117 third-year students, and 101 fourth-year students. Table 1 presents further demographic and descriptive data pertaining to college students.

**Table 1:** Descriptive statistics for the participants (*N* = 487).

**Variables**	**Distribution (*N*)**	**Percentage (%)**
**Age**	487 (M = 20.09)	100 (SD = 1.42)
**Gender**		
Male	246	50.5
Female	241	49.5
**Education level**		
Freshman	159	32.6
Sophomore	110	22.6
Junior	117	24.0
Senior	101	20.7
**Friend number**		
< 50	56	11.5
50–99	108	22.2
100–299	189	38.8
300–499	78	16.0
500–999	35	7.2
≥1000	21	4.3
**Duration**		
< 6 months	11	2.3
6 months−1 year	46	9.4
1–3 years	96	19.7
>3 years	334	68.6
**Time every day**		
< 0.5 h	36	7.4
0.5–1 h	90	18.5
1–2 h	120	24.6
2–3 h	96	19.7
>3 h	145	29.8

### 3.2 Measures

#### 3.2.1 WeChat use intensity

WeChat use intensity was assessed using a three-dimensional framework published by Wen et al. (19), with minor modifications. The following information was collected from the participants: (1) how long has it been since the participants started using WeChat (1 = < 6 months, 2 = 6–12 months, 3 = 12–36 months, 4 = longer than 36 months); (2) how long does it take for the participants to use WeChat every day (1 = < 0.5 h, 2 = about 0.5 to 1 h, 3 = about 1 to 2 h, 4 = about 2 to 3 h, and 5 = longer than 3 h); (3) the number of WeChat friends (1 = < 50, 2 = 50–99, 3 = 100–299, 4 = 300–499, 5 = 500–999, 6 = more than 1000). The researchers employed principal component analysis and factor rotation techniques to examine the duration and timing of participants' usage of the WeChat platform. The values were ultimately standardized to a scale of 0–100, resulting in the creation of the variable representing the intensity of WeChat usage. The Cronbach's alpha coefficient for this scale was determined to be 0.606, a value that is within an acceptable range to conduct exploratory research (75).

#### 3.2.2 Social use of WeChat

The measurement of the social use of WeChat was conducted using four questions that were adapted from the study conducted by Chang and Zhu (40). The participants were queried regarding the extent to which they agreed with statements pertaining to several aspects of WeChat usage, including “meeting new friends through WeChat,” “maintaining communication with friends via WeChat,” “keeping in touch with teachers and classmates through WeChat,” and “accomplishing academic tasks through WeChat.” Participants were requested to provide their feedback using a 5-point Likert scale, ranging from 1 (“strongly disagree”) to 5 (“strongly agree”). The cumulative values of four items were aggregated to assess the extent of social utilization of WeChat. The Cronbach's alpha coefficient for this scale was determined to be 0.710.

#### 3.2.3 Entertainment use of WeChat

The researchers employed the revised three-item scale created by Guo et al. (34) to assess the extent of entertainment utilization of WeChat. The aforementioned projects encompass the examination of activities on the WeChat platform, the utilization of WeChat for entertainment purposes, and the engagement with WeChat as a means of passing time. The participants were also requested to provide their reaction using a 5-point Likert scale, ranging from 1, indicating “strongly disagree,” to 5, indicating “strongly agree.” The scale's Cronbach's alpha coefficient in this study was 0.819.

#### 3.2.4 Bonding social capital

The researchers utilized the modified bonding social capital scale, established by Ellison et al. (76), to evaluate bonding social capital. The items include “I believe some friends can help me solve problems through WeChat” and “WeChat friends can help me solve the unfair things I have encountered.” Participants expressed their agreement on the six questions, using a 5-point Likert scale (1 = “strongly disagree” to 5 = “strongly agree”). Higher scores indicate higher levels of bonding social capital. These scores were later summed to measure bonding social capital. Cronbach's alpha of the bonding social capital scale was 0.839.

#### 3.2.5 Life satisfaction

The Satisfaction with Life Scale (SWLS), developed by Diener et al. (65), was utilized to assess individuals' levels of life satisfaction. The scale comprises five items, including statements such as “my life is roughly in line with my ideals” and “my life is very satisfactory.” Participants were instructed to assess the degree to which they agreed with the statements using a 7-point Likert scale, ranging from 1 (strongly disagree) to 7 (strongly agree). The summation of scores for all items was conducted to assess the comprehensive measure of life satisfaction. The higher the score, the greater the level of life satisfaction. The present study yielded a Cronbach's alpha coefficient of 0.908 for this scale.

#### 3.2.6 Loneliness

The measurement of loneliness was conducted using the social loneliness subscale of the Emotional vs. Social Loneliness Scale [ESLS; (77)]. The ESLS comprises two distinct subscales, namely the social loneliness subscale and the emotional loneliness subscale. According to Russell et al. (78), social loneliness refers to the subjective experience of not having sufficient companionship, whereas emotional loneliness refers to the subjective experience of not having sufficient affectionate relationships. The social loneliness subscale is deemed more appropriate for the goal of conducting research. Participants were instructed to assess the caliber of the social encounter for a set of five items using a Likert scale ranging from 1 (indicating never) to 5 (indicating always). The questionnaire comprises three statements, namely, “it seems that everyone around me is a stranger,” “I can be part of a circle of friends and become one of them,” and “I cannot get much satisfaction from the group I participated in.” The range of total scores spans from 5 to 25 points. The higher the score is, the stronger the loneliness. In this study, Cronbach's alpha of the questionnaire was 0.675.

#### 3.2.7 Control variables

The demographic variables, including age, gender, and educational level, were controlled in accordance with prior studies (48, 79, 80).

### 3.3 Data analytical strategy

The analysis was performed sequentially, consisting of three distinct processes, utilizing the statistical software SPSS version 22. First, Microsoft Excel was employed to perform data cleansing and processing procedures on the raw data. Second, this study employed preliminary statistics and conducted an assessment for common method variance (CMV) utilizing SPSS 22.0. Furthermore, to evaluate the robustness of this measurement model, an analysis is conducted to test the reliability and validity of the constructs employed in the current study. Third, the presentation included descriptive statistics. This study employed correlation analysis to investigate the associations of WeChat use intensity, WeChat use motivations, bonding social capital, and mental health. To conduct a more comprehensive examination of the mediation impacts of WeChat use incentives, including social use and entertainment use, as well as bonding social capital, the bias-corrected bootstrap approach was employed. The methodology employed in this study utilizes an alternative sampling technique, whereby each individual instance has the potential to be selected numerous times to generate multiple samples (81). This strategy effectively mitigates the bias that may arise from a non-normal sampling distribution. The study employed Model 6 in the SPSS macro PROCESS v3.0 plugin to examine the sequential mediation effects. The present study employed a methodological approach to examine the mediating effect, utilizing the estimation of a bootstrap 95% confidence interval (CI) over 10,000 re-sampling iterations. According to Hayes (81), if the 95% CI does not include the value of 0, it indicates that the mediation effect is statistically significant.

## 4 Results

### 4.1 Common method bias tests

To mitigate the potential influence of common method bias, various procedural measures were employed. These efforts included the employment of anonymous personal information and the deliberate reversal of specific components of the projects throughout the data gathering phase. In addition, the utilization of Harman's single-factor test is employed to assess the existence of common method biases, as outlined by Podsakoff et al. (82). The results suggest that there exist eight common factors with feature roots above a value of one. The initial shared component, namely, accounts for 35.82% of the total variation, which is below the critical criterion of 40%. Therefore, it can be inferred that the presence of common technique bias is absent.

### 4.2 Bivariate correlations

Before examining the hypothesis, preliminary descriptive and correlational analyses are performed. The results of the descriptive statistics and correlation matrix are presented in Table 2. The findings presented in Table 2 demonstrate a significant positive association between the level of WeChat usage and its utilization for social purposes (*r* = 0.307, *p* < 0.001), entertainment use (*r* = 0.223, *p* < 0.001), and the development of bonding social capital (*r* = 0.227, *p* < 0.001). Additionally, a noteworthy negative correlation is observed between WeChat usage and feelings of loneliness (*r* = −0.253, *p* < 0.001). Moreover, a negative association has been observed between all four aforementioned qualities and the experience of loneliness. Furthermore, the study revealed that the utilization of social WeChat and entertainment WeChat had a notable positive impact on the cultivation of bonding social capital (*r* = 0.409, *p* < 0.001; *r* = 0.384, *p* < 0.001, respectively).

**Table 2:** Means, SD, and zero-order correlations for all the key study variables (*N* = 487).

**Variables**	**Mean**	**SD**	**1**	**2**	**3**	**4**	**5**	**6**	**7**	**8**	**9**
Age	20.09	1.423	1								
Gender level	0.505	0.500	−0.018	1							
Education	2.33	1.136	−0.021	−0.136^***^	1						
WUI	59.343	20.534	−0.085^*^	0.061	0.061	1					
SUW	14.672	3.596	0.087^*^	−0.061	−0.071	0.307^***^	1				
EUW	10.062	3.334	−0.062	−0.043	−0.090^**^	0.223^***^	0.601^***^	1			
BSC	19.713	3.344	0.014	−0.023	−0.094^**^	0.227^***^	0.409^***^	0.384^***^	1		
LS	23.273	6.821	0.107^**^	−0.094^**^	−0.061	0.069	0.457^***^	0.496^***^	0.400^***^	1	
LL	12.546	3.188	0.076^*^	0.060	−0.079^*^	−0.253^***^	−0.268^***^	−0.219^***^	−0.451^***^	−0.311^***^	1

^*^p < 0.1, ^**^p < 0.05, ^***^p < 0.01.

### 4.3 Serial-multiple mediation model outcomes

This study employed four serial-multiple mediation models to examine the relationships between WeChat use intensity, social use of WeChat, bonding social capital, life satisfaction, and loneliness. Specifically, the study investigated the mediation roles of the social use of WeChat and bonding social capital in the association between WeChat use intensity and life satisfaction, as well as the mediating roles of the social use of WeChat and bonding social capital in the effect of WeChat use intensity on loneliness. Additionally, the study explored the mediation roles of entertainment use of WeChat and bonding social capital in the effect of WeChat use intensity on life satisfaction, as well as the mediation roles of entertainment use of WeChat and bonding social capital in the effect of WeChat use intensity on loneliness. The present study utilized four serial-multiple mediation models to investigate the associations among the intensity of WeChat use, the social use of WeChat, bonding social capital, life satisfaction, and loneliness. The present study aimed to examine the mediating effects of the social use of WeChat and bonding social capital in the relationship between WeChat use intensity and life satisfaction. Additionally, this study sought to investigate the mediating effects of the social use of WeChat and bonding social capital in the impact of WeChat use intensity on feelings of loneliness. Furthermore, the research examined the mediating effects of WeChat's entertainment use and bonding social capital on the relationship between the intensity of WeChat use and life satisfaction. Additionally, it investigated the mediating effects of WeChat's entertainment use and bonding social capital on the relationship between the intensity of WeChat use and feelings of loneliness. Age, gender, and educational level were all controlled in the four models.

#### 4.3.1 WeChat use intensity, social use of WeChat, bonding social capital, and mental health

**Figure 2:**
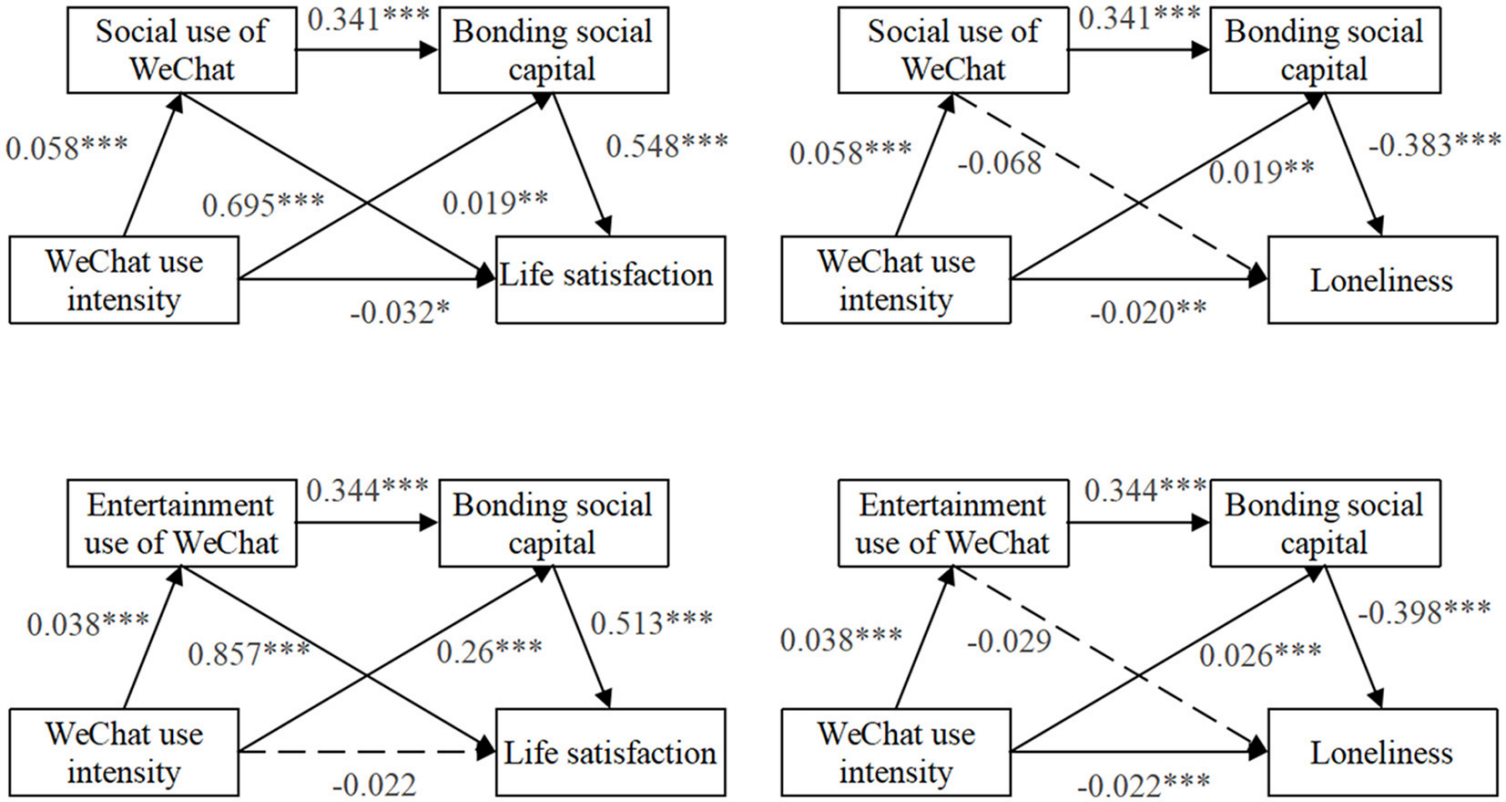
Serial mediation model shows effects of WeChat use intensity, WeChat use motivations and bonding social capital on mental health. Standardized regression coefficients were obtained after controlling for age, gender, and education level. *p < 0.05; **p < 0.01; ***p < 0.001.

The present study aimed to investigate the correlation between the intensity of WeChat usage and life satisfaction, while also exploring the potential mediating effects of the social use of WeChat and bonding social capital. The results shown in Figure 2 indicate that there was a significant relationship between the intensity of WeChat use and life satisfaction (β = −0.032, *p* < 0.05). Furthermore, the 95% confidence interval (CI) for the relationship between WeChat use intensity and life happiness, namely, through the social use of WeChat, did not include the value of 0 (CI = [−0.059; −0.005], as shown in Table 3). The intensity of WeChat usage has an impact on life satisfaction in three distinct manners, as outlined in Model 1. The impact of WeChat usage intensity on life happiness is mediated by the social use of WeChat. The findings of the study indicated a significant positive relationship between the intensity of WeChat usage and the social use of WeChat (β = 0.058, *p* < 0.001). The 95% confidence interval (CI) for the relationship between the intensity of WeChat use and life satisfaction, namely, through the social use of WeChat, did not include the value of 0 (CI = [0.026; 0.056], as shown in Table 3). Furthermore, the utilization of WeChat has been found to have an impact on individuals' life satisfaction by influencing the strength of their bonding social capital. The findings of the study indicate a significant positive correlation between the use of WeChat and the development of bonding social capital (β = 0.695, *p* < 0.001). Furthermore, the study reveals a positive association between bonding social capital and life satisfaction (β = 0.548, *p* < 0.001). In addition, the 95% confidence interval (CI) for the relationship between WeChat use intensity and life satisfaction through bonding social capital did not include the value of 0 (CI = [0.003; 0.020], as shown in Table 3). Third, the intensity of WeChat usage has an impact on individuals' life satisfaction, specifically through the social use of WeChat and the development of bonding social capital. The findings of the study indicate a significant positive association between the utilization of WeChat for social purposes and the development of bonding social capital (β = 0.341, *p* < 0.001). Furthermore, the 95% confidence interval (CI) for the relationship between WeChat usage intensity and life satisfaction, namely through the social use of WeChat and bonding social capital, did not include the value of zero (CI = [0.006; 0.016], as shown in Table 3). According to the research conducted by MacKinnon et al. (83) and Tzelgov and Henik (84), the appearance of a suppression effect can be observed within a mediation model when the direct and mediated effects of the independent variable on the dependent variable have opposing signs. The potential impact of utilizing WeChat lies in its ability to alleviate the negative effects connected with its usage on individuals' overall life satisfaction. This can be achieved through the promotion of social use motivation and the strengthening of bonding social capital. Hence, the utilization of WeChat for entertainment purposes exerts a suppressive effect on the association between the intensity of WeChat usage and life satisfaction.

**Table 3:** Summary of serial-multiple mediation results between WeChat use intensity and mental health (*N* = 487).

	**Effect**	**BootSE**	**95% CI**	
			**BootLLCI**	**BootULCI**
Direct effect of WUI on LS	−0.032	0.014	−0.059	−0.005
**Indirect effect of WUI on LS**				
WUI → SUW → LS	0.040	0.008	0.026	0.056
WUI → BSC → LS	0.011	0.004	0.003	0.020
WUI → SUW → BSC → LS	0.011	0.002	0.006	0.016
Total effect of WUI on LS	0.061	0.009	0.044	0.080
Direct effect of WUI on LL	−0.020	0.007	−0.033	−0.007
**Indirect effect of WUI on LL**				
WUI → SUW → LL	−0.004	0.0023	−0.009	0.001
WUI → BSC → LL	−0.007	0.003	−0.014	−0.002
WUI → SUW → BSC → LL	−0.008	0.002	−0.011	−0.005
Total effect of WUI on LL	−0.019	0.004	−0.027	−0.012
Direct effect of WUI on LS	−0.022	0.013	−0.048	0.003
**Indirect effect of WUI on LS**				
WUI → EUW → LS	0.032	0.008	0.002	0.048
WUI → BSC → LS	0.013	0.005	0.006	0.023
WUI → EUW → BSC → LS	0.007	0.002	0.003	0.011
Total effect of WUI on LS	0.052	0.009	0.034	0.070
Direct effect of WUI on LL	−0.022	0.007	−0.035	−0.009
**Indirect effect of WUI on LL**				
WUI → EUW → LL	−0.001	0.002	−0.005	0.003
WUI → BSC → LL	−0.010	0.003	−0.017	−0.005
WUI → EUW → BSC → LL	−0.005	0.002	−0.009	−0.002
Total effect of WUI on LL	−0.017	0.004	−0.024	−0.009

CI, Confidence interval; WUI, WeChat use intensity; LS, Life satisfaction; SUW, Social use of WeChat; EUW, Entertainment use of WeChat; BSC, Bonding social capital; LL, Loneliness.

Second, the present study examined the second mediation model, specifically focusing on the mediating role of the social use of WeChat and bonding social capital in the relationship between the intensity of WeChat use and feelings of loneliness. The results from Model 2, as depicted in Figure 2, indicate a strong negative association between the usage intensity of WeChat and feelings of loneliness (β = −0.020, *p* < 0.01). The initial findings indicate that there was no significant mediation impact of WeChat use intensity on loneliness through the social use of WeChat. The study found that there is no significant direct relationship between the intensity of social usage of WeChat and feelings of loneliness (β = −0.068, *p* > 0.05). The 95% confidence interval (CI) for the relationship between WeChat use intensity and loneliness, as measured by the social use of WeChat, ranged from −0.009 to 0.001 (CI = [−0.009; 0.001], see Table 3). Additionally, our study revealed a substantial mediation impact of bonding social capital in the relationship between the intensity of WeChat use and feelings of loneliness. The statistical analysis reveals a strong relationship between the intensity of WeChat use and the development of bonding social capital (β = 0.019, *p* < 0.01). The study found a statistically significant negative relationship between bonding social capital and loneliness (β = −0.383, *p* < 0.001). The results of the bootstrapping analysis revealed that bonding social capital had a significant indirect influence on the relationship between WeChat use intensity and loneliness. The coefficient for this indirect effect was −0.007, with a 95% confidence interval ranging from −0.014 to −0.002. These findings are presented in Table 3. Furthermore, it was shown that there was a significant direct effect of the intensity of WeChat use on the social use of WeChat (β = 0.058, *p* < 0.001). Furthermore, it is noteworthy that the impact of utilizing WeChat for social purposes on the development of bonding social capital was shown to be statistically significant (β = 0.341, *p* < 0.001). The relationship between the intensity of WeChat use and feelings of loneliness was found to be mediated by the serial pathway of WeChat use intensity leading to social use. The study found a significant negative relationship between the use of WeChat and feelings of loneliness, as shown by the confidence interval of −0.011 to −0.005 (95% CI), as shown in Table 3.

#### 4.3.2 WeChat use intensity, entertainment use of WeChat, bonding social capital, and mental health

Third, this study examined the relationship between WeChat use intensity and life satisfaction with the mediation roles of entertainment use of WeChat and bonding social capital. As shown in Figure 2 Model 3, WeChat use intensity was not significantly associated with life satisfaction (β = −0.022, *p* > 0.05). The direct effect of WeChat use intensity on entertainment use of WeChat was significant (β = 0.038, *p* < 0.001). The direct effect of the entertainment use of WeChat on life satisfaction was significant (β = 0.344, *p* < 0.001). In addition, the 95%CI of WeChat use intensity to life satisfaction through entertainment use of WeChat does not contain 0 (IC = [0.002, 0.048, see Table 3]), indicating that the mediation effect of entertainment use on the relationship between WeChat use intensity and life satisfaction was significant. Furthermore, the direct effect of WeChat use intensity on bonding social capital was significant (β = 0.26, *p* < 0.001). The direct effect of bonding social capital on life satisfaction is significant (β = 0.513, *p* < 0.001). The 95% CI of WeChat use intensity to life satisfaction through bonding social capital did not contain 0 (IC = [0.006, 0.023, see Table 3]), indicating that the mediation effect of bonding social capital was significant. As shown in Model 3, the entertainment use of WeChat was significantly associated with bonding social capital (β = 0.344, *p* < 0.001). In addition, the 95% CI of WeChat use intensity to life satisfaction through both entertainment use of WeChat and bonding social capital did not contain 0 (IC = [0.003, 0.011, see Table 3]). Thus, in model 3, WeChat use intensity has effects on life satisfaction in three ways (WeChat use intensity → entertainment use WeChat → life satisfaction, WeChat use intensity → bonding social capital → life satisfaction, and WeChat use intensity → entertainment use of WeChat → bonding social capital → life satisfaction; see Model 3).

Finally, this study tested the fourth mediation model: the mediation effects of entertainment use of WeChat and bonding social capital on the effect of WeChat use intensity and loneliness. As shown in Figure 2 Model 4, WeChat use intensity was significantly associated with loneliness (β = 0.038, *p* < 0.01). First, we found that the mediation effect of WeChat use intensity on loneliness through entertainment use of WeChat was not significant. WeChat use intensity was positively associated with entertainment use of WeChat (β = 0.038, *p* < 0.001). However, the direct effect of bonding social capital on loneliness was not significant (β = −0.029, *p* > 0.05). The results of bootstrapping also revealed that the entertainment use of WeChat (coefficient = −0.001, 95% CI [−0.005, 0.003]) was not a significant mediator of the relationship between WeChat use intensity and loneliness (see Table 3). Second, the mediation effect of WeChat use intensity on loneliness through bonding social capital was significant. The direct effect of WeChat use intensity on bonding social capital was significant (β = 0.026, *p* < 0.001). Bonding social capital was reported to be significantly and negatively associated with loneliness (β = −0.398, *p* < 0.001). In addition, the indirect effect from bootstrapping also revealed that bonding social capital (coefficient = −0.010, 95% CI [−0.017, −0.005]) was a significant mediator of the relationship between WeChat use intensity and loneliness (see Table 3). Third, the direct effect of the entertainment use of WeChat on bonding social capital was significant (β = 0.344, *p* < 0.001). In addition, the 95%CI of WeChat use intensity to loneliness through the serial-multiple mediations of entertainment use of WeChat and bonding social capital did not contain 0 (IC = [−0.009; −0.002, see Table 3]). Therefore, the indirect impacts of WeChat use intensity on loneliness exist in two ways in model 4: WeChat use intensity → bonding social capital → loneliness and WeChat use intensity → entertainment use WeChat → bonding social capital → loneliness.

## 5 Discussion

### 5.1 Summary of the key results

Based on the UGT theory and self-determination theory, this study attempted to systematically examine the underlying mechanism between WeChat use intensity and Chinese students' mental health, with a specific focus on WeChat use motivation (social use of WeChat and entertainment use of WeChat) and bonding social capital. The study revealed that the intensity of WeChat usage had significant effects on the degrees of life satisfaction and loneliness among college students in China, both directly and indirectly. Detailed findings are discussed below.

### 5.2 Mediation pathways from WeChat use intensity to mental health through social use of WeChat and bonding social capital

The present study investigated the mediating role of the social use of WeChat and bonding social capital in the relationship between WeChat use intensity and mental health, employing bootstrapping techniques for data analysis. The results indicated that the use of WeChat for social purposes played a mediating role in the relationship between the intensity of WeChat use and individuals' life satisfaction. This finding supports our hypothesis H1a. On the other hand, there was no observed correlation between the utilization of WeChat for social motivation and feelings of loneliness. Thus, the H1b hypothesis was not substantiated. The results of the study indicate a favorable and significant correlation between the intensity of WeChat usage and its social use. The results suggest that a higher level of WeChat usage intensity may enhance individuals' motivation to engage in social interactions, aligning with prior studies that have found a positive association between WeChat usage intensity and social usage motivation (80). Furthermore, there was a favorable correlation between the utilization of WeChat for social purposes and individuals' life satisfaction. The results of the study also suggest that there is no significant relationship between the use of WeChat for social motivation and feelings of loneliness. This finding contradicts the earlier research conducted by Aydin et al. (85), which primarily focused on investigating the relationship between Facebook usage and loneliness. This may explain that the increased intensity of social media usage among college students may be associated with higher levels of social activity and cohesion within online networks, facilitating easier communication and interaction with friends, family, and relatives. Moreover, an increased number of connections can provide Chinese college students with greater options for self-expression and receiving recognition, thereby protecting against feelings of loneliness.

Second, this study indicated that students with high WeChat use intensity tend to experience a high level of bonding social capital, which in turn experienced better life satisfaction and lower loneliness. Therefore, H3a and H3b were supported. Previous research has also confirmed bonding social capital mediated the association between WeChat use intensity and mental health (18). Burke et al. (71) also demonstrated that low bonding social capital is significantly associated with higher loneliness. The finding may be explained by the psychological compensation theory (86). Online WeChat socialization can help people overcome the embarrassment and inconvenience of face-to-face communication, especially for introverted students. Additionally, in an era of fast-moving and modern society, with the acceleration of life pace, friends and family usually live in different places. WeChat, as an instant and real-time communication platform, can break through geographical restrictions, help keep in touch with social relationships, and seek psychological compensation (87), thereby reducing loneliness (18). However, the results of this study are inconsistent with the study by Hou et al. (88) that excessive use of WeChat had a negative impact on Chinese college students' loneliness. This is possibly because when college students mainly use WeChat at moderate intensity, which increases the opportunities for communication and self-presentation with others, it helps to obtain emotional support from family and friends (80). Furthermore, it is noteworthy to observe that there exist significant distinctions between the intensity of WeChat usage and the occurrence of excessive WeChat usage. The term “excessive use of WeChat” pertains to the psychological manifestation characterized by the intensity of psychological symptoms, including emotional fluctuations, anxiety, and interpersonal conflicts, resulting from the utilization of WeChat. Furthermore, Gao et al. (89) have established a correlation between the two variables, suggesting that the intensity of WeChat usage can serve as an indicator of excessive use of the platform.

Third, the findings of this study's serial mediation effects supported H4a and H4b, that is, students with high WeChat use intensity were sequentially linked with increased WeChat social use first and then improving their bonding social capital, which was, in turn, related to improvement in life satisfaction and decreased loneliness. The findings correspond with the studies that suggest SNS (especially WeChat) is becoming an indispensable part of personal daily life, which provides convenience for expanding the scope of contacts and facilitating social connections and social ties with other members (80, 90).

### 5.3 Mediation pathways from WeChat use intensity to mental health through entertainment use of WeChat and bonding social capital

This study conducted two serial mediation models to examine the mediators of entertainment use of WeChat and bonding social capital of the relationship between WeChat use intensity and life satisfaction and loneliness. First, the results of this study demonstrated that WeChat use intensity positively influenced students' entertainment usage motivation. This result is consistent with previous research results that social media contributes to relaxed use and time pass (91). Second, this study found that the more entertainment usage of WeChat, the better satisfaction with life. Therefore, this study proved H2a. The research indicated that the entertainment use of WeChat is not related to loneliness, which aligns with the findings of a longitudinal study conducted by Dienlin et al. (92). Hence, H2b is not verified. One possible explanation could be that the effects of media usage are contingent upon how individuals engage with it, rather than the specific type of media being consumed (21). This is because users can actively pursue and attain gratification in accordance with their personal objectives and motivations. In other words, various forms of media, such as WeChat, offer individuals a means to engage in fulfilling interpersonal interactions (93). WeChat has rich interactive functions such as chat, red envelope (hongbao), the circle of friends, “Like,” and comments facilitating interpersonal communication (4, 94). It fully meets the communication needs of college students. Meanwhile, due to the traditional Chinese implicit culture, the use of social media can help students express their inner feelings that cannot be expressed face to face. Individuals frequently select captivating forms of entertainment, such as films, music, games, and the like, as a means to alleviate their perception of existential futility. Hence, the utilization of WeChat for recreational purposes does not exert any influence on the loneliness experienced by its users.

This study proved that the entertainment use of WeChat was positively associated with bonding social capital, which is inconsistent with a study by Guo et al. (34). Guo et al. (34) held that the entertaining use of social media might reduce the perception of social capital. The possible reason may be because Guo et al. (34) subjects are international students who are vulnerable to academic stress, acculturation stress, and mental diseases which are salient predictors of addictive smartphone behavior (95, 96). The main reason may be explained that the utilization of WeChat for entertainment purposes does not inherently result in addiction and bad effects. In contrast, an increasing number of college students are utilizing applications such as WeChat games as a means to comprehend and reinforce preexisting social connections. Hence, the significance of the utilization motive may not be paramount; rather, its relevance lies in its alignment with internal requirements and its facilitation of the establishment of bonding social capital. Third, the findings of the serial mediation supported the hypothesis of H5a and H5b. Individuals who use WeChat for entertainment more frequently experience a higher level of bonding social capital, which in turn leads to greater life satisfaction and lower loneliness. Corresponding with the theoretical and empirical studies of social capital theory (50), SNS use for entertainment can help individuals make new channels to have leisure and recreational activities with friends (97), which can strengthen “strong tie” for users (98) and reduce their interpersonal distress (11).

In summary, social use of WeChat use motivation and entertainment use of WeChat use motivation both acted as a mediating effect on WeChat use intensity and mental health. It is imperative to not only ascertain the temporal aspects, frequency, and motivation of an individual's utilization of WeChat daily but also to comprehensively comprehend the nature of activities exhibited at the personal level, and to evaluate the potential influence on one's mental health.

### 5.4 Limitations and suggestions for future study

Various restrictions in the current research should be clearly addressed in future research. First and foremost, the interviewers were drawn from a single city, and the sample consisted primarily of young adults with an average age of 20.09 years. Furthermore, this research was constrained by money and time constraints, which, if expanded, would have considerably enlarged the sample size and thus improved the external validity of the research. Therefore, it is necessary to be cautious in extending the research results to other populations. Future research is recommended to further expand sample groups in different cultural-geographical circumstances and improve the test efficiency, making the findings more robust and persuasive. Second, due to the cross-sectional nature of the survey, the study did not establish a causal relationship among the main variables, including WeChat use intensity, WeChat use motivation, bonding social capital, and mental health. Longitudinal studies and experimental methods should be conducted further to investigate causal relationships among them in subsequent research. Third, it is worth considering the potential enrichment of the study model's dimensions. Future studies may benefit from incorporating individuality qualities, such as introversion or extroversion. Future studies might further investigate additional aspects of WeChat usage motives to examine their possible impact on mental wellbeing. Moreover, it is imperative to explore the psychological framework that is closely associated with Chinese culture to have a thorough understanding of the use of WeChat by individuals from China and other Asian nations. The concepts referred to include guānxì, which refers to interpersonal relationships, and mianzi, which pertains to face or social reputation. Furthermore, it is anticipated that the use of this methodology aimed at broadening the scope of research will yield more comprehensive and insightful findings. Ultimately, it is imperative to show prudence when extrapolating the conclusions of our investigation to diverse academic or social contexts, as well as to alternative social networking platforms.

## 6 Conclusion

The current study was the first to explore the relationship between WeChat use intensity and mental health among Chinese college students using the serial-multiple mediation model. This article finds that WeChat use intensity had both direct and indirect impacts on the levels of life satisfaction and loneliness experienced by college students in China. Specifically, the utilization of WeChat for social motivation and entertainment motivation was found to have a suppressive effect on the relationship between the intensity of WeChat usage and individuals' life satisfaction. The association between the intensity of WeChat usage and mental health outcomes (life satisfaction and loneliness) was found to be mediated by bonding social capital. Furthermore, the association between the intensity of WeChat usage and mental health was found to be mediated by the sequential mediation effects of using WeChat for social motivation and bonding social capital, as well as the sequential mediation effects of using WeChat for entertainment motivation and bonding social capital. The present study not only confirmed the suitability of the self-determination theory and the UGT theory for the usage of SNS but also provided insights into potential intervention strategies for Chinese adolescents.

From a theoretical viewpoint, this research has made several significant contributions to the studies of WeChat use and mental health. First, a large body of theoretical and empirical studies has examined the relationship among SNS utilization, SNS use motivations, social capital, and mental health (19, 27, 34, 41). However, no studies have examined the serial mediation effects of motivations and bonding social capitals on the relationship between WeChat use intensities and mental health. The current study contributed to clarifying the indirect mechanisms of how time spent on WeChat leads to better mental health. The obtained results of this research firstly discovered the support of the chained mediation role of two types of WeChat use motivations and bonding social capital on the relationship between WeChat use intensity and mental wellbeing, which shed light on the research of WeChat's impacts on subjective wellbeing.

Second, the current research also highlighted some practical implications. The results show that the time spent on WeChat can improve the mental health of college students. This may be because the use of WeChat provides convenience for college students' social interaction, meets their social and entertainment needs, and strengthens the social capital of college students. Based on the findings, it is suggested that proper and scientific use of new media will exert positive effects on college students' psychological wellbeing. Therefore, policymakers and practitioners are recommended to develop new perspectives on how WeChat use intensity affects mental health. Finally, specific online activities that not only meet social and recreational gratifications but can promote students' social capital are highly recommended for college students.

## Data availability statement

The original contributions presented in the study are included in the article/supplementary material, further inquiries can be directed to the corresponding author.

## Ethics statement

Ethical review and approval was not required for the study of human participants in accordance with the local legislation and institutional requirements. Written informed consent from the patients/participants was not required to participate in this study in accordance with the national legislation and the institutional requirements.

## Author contributions

MX and JL drafted and conducted the manuscript. MX contributed to the processes of modeling, data analysis, original writing, revision, proofreading, and finalizing of the manuscript. JL contributed to the revision, proofreading, and finalizing of the manuscript. All authors contributed to the article and approved the submitted version.
